# A Survey of Current Knowledge on Sexually Transmitted Diseases and Sexual Behaviour in Italian Adolescents

**DOI:** 10.3390/ijerph13040422

**Published:** 2016-04-13

**Authors:** Francesco Drago, Giulia Ciccarese, Francesca Zangrillo, Giulia Gasparini, Ludovica Cogorno, Silvia Riva, Sanja Javor, Emanuele Cozzani, Francesco Broccolo, Susanna Esposito, Aurora Parodi

**Affiliations:** 1 Di.S.Sal. Department of Dermatology, IRCCS Azienda Ospedaliera Universitaria San Martino-IST, Genoa 16132, Italy; frdrago@libero.it (F.D.); gasparini.giulia@yahoo.it (G.G.); lucogorno@yahoo.it (L.C.); silvia.riva@ymail.com (S.R.); javor.med@gmail.com (S.J.); emanuele.cozzani@unige.it (E.C.); aurorap@unige.it (A.P.); 2 Di.S.Sal. Department of Health Sciences, IRCCS Azienda Ospedaliera Universitaria San Martino-IST, Genoa 16132, Italy; francesca.zangrillo@live.it; 3 Department of Health Sciences, University of Milano-Bicocca, Monza 20900, Italy; broccolof@gmail.com; 4 Pediatric Highly Intensive Care Unit, Department of Pathophysiology and Transplantation, Università Degli Studi di Milano, Fondazione IRCCS Ca’ Granda Ospedale Maggiore Policlinico, Milan 20122, Italy; susanna.esposito@unimi.it

**Keywords:** sexually transmitted diseases, sexual behaviour, adolescents, sex education, sexual health

## Abstract

Worldwide, 500 million people a year acquire a sexually transmitted disease (STD). Adolescents, accounting for 25% of the sexually active population, are the most affected. To analyze sexual behavior among Italian adolescents and their knowledge of STDs, with the goal of preventing their transmission, a questionnaire was administered to 2867 secondary school students (1271 males and 1596 females) aged 14–21 years. For the study, 1492 students were interviewed in Genoa (Northern Italy) and 1375 in Lecce (Southern Italy). For 37% of the respondents, parents and teachers were the main source of information on sex, and 95% believed that school should play the primary role in sex education. However, only 9% considered the sex education they received in school good. Noteworthy, only 0.5% of the teenagers recognized the sexually transmitted diseases from a list of diseases, and 54% of them did not know what a Pap test was. Confusion about the meaning of contraception and prevention was evident; only 22% knew that condoms and abstinence are the only methods for preventing STDs. Finally, a consistent number of students are exposed to risk factors for STDs transmission; e.g., alcohol and recreational drug use, promiscuity and improper condom use. On the basis of our study, there is an urgent need for the introduction of sex education as a proper subject in Italian schools.

## 1. Introduction

Sexually transmitted diseases (STDs) are a group of infectious diseases caused by viruses, bacteria, fungi, parasites, protozoa or arthropods that are generally acquired by sexual contact. STDs include more than 30 different conditions, among which the most common are gonorrhoea, chlamydial infection, syphilis, trichomoniasis, chancroid, genital herpes, genital warts, human immunodeficiency virus (HIV) infection and hepatitis B [1].

STDs represent one of the most serious public health issues in the world, in both industrialized and developing countries. The World Health Organization (WHO) estimates that worldwide more than 1 million people acquire a sexually transmitted infection every day and 500 million people each year [1]. Adolescents, although accounting for only 25% of the sexually active population, are the most affected, as they represent almost 50% of all newly acquired STD cases [2]. In Italy, 19.5% of all new cases of STDs are diagnosed in young people (15–24 years old) [3]. Recent studies of sexual activity in Italian adolescents found that many of them have their first sexual experience at a very early age (15.6 ± 1.6 years old), often without protection against STDs [4]. Furthermore, a large percentage has multiple partners [5].

On the basis of these considerations, we asked adolescents in Italian secondary schools to complete a written questionnaire inquiring about their knowledge of STDs, their sexual behaviors and their use of STD prevention methods. Our goals were first to establish the level of knowledge about STDs in Italian adolescents and, secondly, in the meantime, to increase, at least partially, their knowledge on these topics.

## 2. Methods

All subjects gave their informed consent for inclusion before they participated in the study. The study was conducted in accordance with the Declaration of Helsinki, and the protocol was approved by the Ethics Committee of Genoa (Project identification code: 1256).

Our group developed a written questionnaire that comprised 39 questions divided into three sections concerning the social context of the students (family, friends, school and religion) (Section 1), knowledge about STDs (Section 2) and the adolescent’s behavior (Section 3), as shown in Table 1. The questionnaire was presented to the students in Italian.

Northern and southern Italian schools from the Liguria and Puglia regions (respectively, from the cities of Genoa and Lecce) were selected. We included all types of secondary school: high schools (classic, scientific, linguistic, psycho-pedagogical and artistic high schools) and vocational schools (technical and professional institutes) to ensure that the sample was as heterogeneous as possible.

After having contacted by phone and/or e-mail the headmaster of each school, we planned one or more meetings (depending on the number of the involved students) at every institute to propose and deliver the questionnaire and to comment on it after the compilation. Because approximately 45% of the students were under 18 years old, the teachers requested parental consent to join the meeting and they always agreed. After a brief introduction on the survey purpose, we motivated the students to give only genuine answers since the questionnaires remained anonymous. The questionnaires were administered to the students in their own classes where at least two of our group and one teacher were present and available to explain the questions. Self-administration of the questionnaire took approximately 20 min. After the compilation of the questionnaire, we provided students with the correct answers and held a brief lecture on STDs and prevention methods. We spent approximately 60 min in each class.

After the questionnaires were administered, the sample was stratified to evaluate possible differences in the answers based on age, gender, type of school and social background. Statistical analysis was performed using the software SPSS Statistics (version 22.0 2013) (IBM Corp., Armonk, NY, USA), the programs Openepi (version 2.3.1) (www.OpenEpi.com) and Excel (version 2011 14.1.3) (Microsoft Corporation, Redmond, WA, USA). The statistical level of significance was assumed as *p* = 0.05.

## 3. Results

From April 2013 to June 2014, a total of 2880 students in 21 different secondary schools in Italy (16 schools in the city of Genoa and five schools in the city of Lecce) were invited to participate in the study. In both cases the schools were located in a urban, not rural, setting. Only 13 students declined. Among the 2867 surveyed subjects, 1271 were males and 1596 were females, with an average age of 17 years (ranging from 14 to 21 years). Among these, 25% of the students attended professional schools, 22% attended technical institutes and 53% attended high schools (23% scientific, 19% classic, 6% linguistic, 4% psycho-pedagogical, 1% artistic high schools). Regarding religion and family, 73.3% (95% CI: 71.78–75.01) of the adolescents declared themselves Catholic; the others were atheist (21.4%), Protestant (0.7%), Orthodox (0.5%), Muslim (1%), or Jewish (0.2%). The influence of religion on sexual choices, as resulted by the questionnaries, played a substantial role in only 16% (95% CI: 14.66–17.34) of cases.

The most interesting results obtained from the first section of the questionnaire concern the sources of sexual information and communication with parents (Supplementary Material, Figure S1). In 37% of cases (95% CI: 35.23–38.77), information about sex came from parents and teachers, in 25% (95% CI: 23.03–26.18) from friends, in 15% (95% CI: 14.18–16.82) from the Internet, in 16% (95% CI: 14.66–17.34) from books and 7% (95% CI: 5.87–7.72) of the students did not want to specify the source of their information. In Genoa, most students (59% compared to only 15% in Lecce; Pearson’s chi-square with Yates’ correction = 581.8; *p* < 0.0000001) received information about sex from their parents and teachers. Conversely, in Lecce, the main sources of information were the Internet (26.5%; 95% CI: 24.17–28.83) and friends (44.5%; 41.87–47.13). Communication with parents was considered excellent by 27% (95% CI: 24.97–28.2) of the students, good by 43% (95% CI: 42–45.63), moderate by 24%, and non-existent by 5%. A substantial difference was observed between Lecce and Genoa: in Lecce, 47% of the students never talked about sex with their parents, whereas this happened regularly in 49% of cases in Genoa. The other results obtained in the first section of the questionnaire are shown in Table 1.

The second section of the questionnaire investigated the actual knowledge of the teenagers about STDs. The most important result in this section concerns the correct knowledge of the diseases that could be sexually transmitted. The subjects were asked to select from among a list of diseases (including HIV, syphilis, hepatitis A, hepatitis B, hepatitis C, herpes simplex infection, candida and genital warts) the ones they believe to be sexually transmitted. Only 15 students (0.5%; 95% CI: 0.24–0.76) answered this correctly. HIV was correctly recognized as a STD by 90% of the students (95% CI: 88.9–91.1), while 65.3% and 46.6% of the respondents correctly recognized syphilis and herpes, respectively. The other results obtained in the second section are shown in Table 2.

The third section of the questionnaire was designed to understand how Italian teenagers approached sex, to assess the risks they are exposed to and also their relationships with their peers and parents. The most notable result of this section is that 61% (95% CI: 59.21—62.79) of the respondents had already had sexual intercourse and 15.5 ± 1.5 years old was the average age of their first sexual intercourse. However, 77% (95% CI: 75.46) of these students used a contraceptive method during the first intercourse (90% condom (95% CI: 88.9—91.1), 4% (95% CI: 3.28—4.72) contraceptive pill and 2.3% (95% CI: 1.75—2.85) both). Stratification revealed that the users were mainly females, older teenagers and high school students. 

Most students were currently using contraceptive methods: 37.3% (95% CI: 35.52—39.07) condoms, 6.5 (95% CI: 5.6—7.4) contraceptive pill, 2.1 (95% CI: 1.58—2.61) both condom and contraceptive pill, while 41% (95% CI: 39.2—42.8) were not currently using contraceptive methods. Regarding other risk factors for STD transmission, 79% (95% CI: 77.51—80.49) of responders admitted using alcohol, especially at parties (58% (95% CI: 56.19—5.81)) and during weekends (22% (95% CI: 20.48—23.52)). In addition, 30% (95% CI: 28.32—31.68) admitted using recreational drugs, cannabis being the most common; LSD, ecstasy and cocaine had been used as well. The other results of the third section of the questionnaire are reported in Table 3.

## 4. Discussion

The World Health Organization defines sexual health as a state of physical, mental and social well-being in relation to sexuality. It requires a positive and respectful approach to sexuality and sexual relationships, as well as the possibility of having pleasurable and safe sexual experiences, free of coercion, discrimination and violence [6]. During a WHO Conference held in Antigua (Guatemala) in May 2000, a panel of experts defined the most important issues regarding sexual health, namely the need for promotion of sexual relationships practiced in safe and responsible manner, and the need to implement the awareness of the risk of contracting and transmitting STDs (including AIDS) [7]. Considering our many years of experience, the few studies conducted on the subject in Italy and the substantial cultural differences between the North and South of the country, we decided to conduct a survey among Italian adolescents to better assess their knowledge about STDs, and their sexual behavior. In building our study, we took examples from similar studies conducted in other countries all over the world [8,9,10].

The results obtained from our survey showed that there is an urgent need for sex education in Italy. Most of the young people surveyed (95%) correctly believed that school should play the most important role in this educational process. Despite this awareness, only 9% of the young respondents rated the sex education they received at school good, while 36% rated it poor, and an astonishing 23% considered it absolutely nonexistent. These data are very alarming and highlighted a substantial dysfunction in the Italian educational system. However, various studies in the literature have revealed similar scenarios in other countries around the world. Rink *et al.*, found that Greenlandic youth do not have adequate information about STDs prevention [9,10,11,12]. In Portugal, as highlighted by the systematic review conducted in 2014 by Mendes *et al.*, less than half of Portuguese teenagers have ever attended classes on reproductive health [13]. In Italy, the first legislative proposal for the introduction of sex education in schools began in the early 1900s, but it was not successful. The latest legislative proposal in Italy dates back to November 1992. Since then, the debate continues, but not much practical has been done. Furthermore, our data indicate a highly significant difference in the sources of information on sexuality between northern and southern Italy. In fact, in Genoa, in 59% of cases, the source of information was family and teachers, while in Lecce only 15% of students reported that they had received information about sex from these sources.

The uniqueness and importance of the parent-child relationship for the prevention of STDs was discussed in 2010 [14] by Deptula *et al.*, who found that a good relationship between parents and children is associated with lower levels of unprotected sex, unwanted pregnancies and STDs in adolescents. Different studies from other Mediterranean Catholic countries have indicated that parent-adolescent communication is crucial for adolescent health indicators [15,16,17]. Studies about the importance of parent-adolescent communication have been recently extended to other countries of the European context showing significant discrepancies with the results obtained in different cultural contexts [15]. However, unfortunately, the WHO documents do not always take into account cultural variations for designing intervention programs.

More than half of Italian adolescents seem to have a good or excellent communication with their parents. Nevertheless, only 7% of them frequently talk with their parents about STDs; most of them talk about this subject with their parents regularly or rarely. In this regard, substantial differences can be noticed between southern and northern Italy: in Lecce, 47% never talk about STDs with their parents, whereas this happens regularly in Genoa (in 49% of cases). This result, together with the differences in the source of information, confirms that there still is a strong difference regarding education and family in different parts of the country. This difference does not necessarily mean that Genoa parents are more acculturated than those of Lecce. A possible explanation could be the fact that talking about sex in a familiar setting in Lecce is considered as a taboo, differently from what happens in Genoa.

Such a profound difference between northern and southern regions was not observed for religion, as would have been expected. In Italy, Catholicism has strong cultural roots and is still taught in Italian schools. Catholicism considers sexuality an absolute taboo, and that sex before marriage or sex that does not result in conception is unacceptable [18]. From what emerges from our study, however, although 73.4% of respondents declared themselves Catholic, only 16% believe that religion has an effective influence on the sexual choices of individuals. More than half of the respondents believe that religion has very little or no influence at all on their sexual choices. Almost one-quarter of the subjects declared themselves atheists. This result was consistent in southern and northern Italy. Religion could be a double-edged sword for teenagers, since abstinence before marriage may protect teenagers from STDs; however, religion may inhibit them and prevent them from talking about STDs freely with parents and teachers [19].

In the second part of the questionnaire, only 0.5% of the students were able to correctly identify the STDs from among a list of diseases. This is particularly troubling because it indicates how helpless young Italians are against STDs that can have serious consequences on their physical and mental health and even on their fertility. The concern is also supported by the fact that only 15.5% of the students knew that STDs can be acquired with any type of partner and that even a single unprotected sexual encounter is sufficient for infection. Furthermore, only 22% had good knowledge about contraceptive and preventive methods. The interviewed teenagers were not able to properly distinguish between preventive methods and contraceptive methods; indeed, among the preventive measures against STDs the students incorrectly indicated the birth control pill, the transdermal patch and *coitus interruptus*.

Regarding the risk of HIV transmission, 37.7% of Genoese students were correct about the vehicle of transmission, but only 14.3% of students from Lecce answered correctly. This evidence clearly indicates that families and education systems, especially in southern Italy, must urgently change. In Italy there have been thousands of new cases of HIV infection since 1982 and the annual incidence is still approximately equal to 6 cases per 100,000 inhabitants. In addition, in 2013, of 3608 new cases, 83.9% were attributable to unprotected sex [20]. Furthermore, it is important to teach young people that there is currently no radical cure for AIDS.

The teenagers we interviewed had inferior knowledge of AIDS compared to HPV infection. Females seemed better informed on HPV infection, as 72.6% affirmed they knew the meaning of the Pap test; older subjects were better informed. However, 46% of respondents claimed to know what the Pap test was, but only 23% were able to describe its function. Moreover, it is very important for adolescents to be aware of the risks of HPV infection; indeed Panatto *et al.* demonstrated that in cervical swabs with normal cytology, the positivity for at least one HPV type was found in 48.1% of women aged 16–17 years, in 15.4% of women aged 18–20 years, in 21.9% of women aged 21–23 years and in 15.5% of women aged 24 to 26 years [21]. Students who attended high schools in Lecce were better informed about HPV infection than their peers who attended vocational schools. Although this difference was not noted among students from Genoa, it underlines the importance of socio-economic background influencing the knowledge of the risks associated with sexual activity.

With regard to primary prevention of STDs, only 17% of respondents knew that there was a vaccine against HPV infections and only 0.1% knew of the existence of the vaccine for hepatitis B. Although ignorance about the existence of the vaccine for hepatitis B does not appear to have negative consequences, as the vaccination has been mandatory in Italy since 1991 [22], the unawareness that there is a vaccine for HPV can have more serious negative repercussions. Indeed, this vaccine is optional in all Italian regions and offered free of charge to all females who have reached 11 years of age. This modality in distribution requires an active involvement of girls and their parents. Compliance is therefore very important and although the National Plan for the Vaccine Prevention 2012–2014 [23] has set as a goal the coverage of at least 95% of eligible subjects, we are still far from this goal.

As far as personal experience, the respondents reported an early sexual debut, with an average age of first intercourse of 15 years. This finding is consistent with the results reported by Panatto *et al.* [4], who conducted a study on the sexual habits of young people in five Italian cities (Genoa, Florence, Turin, Cagliari and Sassari). That study also showed that, in recent years, there has been a tendency to an earlier sexual debut among young Italians. Moreover, 77% of young people in our study reported using contraceptive methods, mainly condoms, during the first sexual intercourse. Those who had used contraceptive methods were more frequently females, older teenagers and high school students. These data are in agreement with the results of Panatto *et al.* It is well known that promiscuity is proportionally related to an increased risk of infection [24], but as far as the respondents in question, only 15% claimed to have had sex with more than two partners in the last two years.

Among the teenagers we interviewed, 46% considered that their friends had a moderate influence on their first sexual intercourse. Peer influence in adolescence is often crucial for sexual behavior [25], and also for alcohol or recreational drug consumption. Alcohol consumption among respondents was high (up to 80%) and 30% admitted to having used recreational drugs. This finding is concerning because both alcohol and recreational drugs, by reducing the ability of judgment, lead to risky behaviors [24,25,26,27,28].

## 5. Conclusions

This study has described the current status of sexual health and reproductive needs of Italian adolescents. On the basis of our data, it is clear that there is an urgent need to introduce sex education as a proper subject in Italian schools. Italian authorities have long neglected this need, therefore failing at least in part to fulfill the Constitution, which states that the Italian Republic shall defend the health of citizens. The European Union also stressed that sex education in Italy has been and is still lacking, putting particular emphasis on political and religious constraints. The European Union hopes that sex education is introduced soon in Italian schools, as it has been for many years already in countries such as Denmark, Sweden, France and Germany [29]. Only by implementing as soon as possible the suggestions made by the European Union will Italy be able to comply with the WHO guidelines. In this regard, our study was not only a survey but also a small contribution to the improvement of sexual health in Italy.

We believe that all types of secondary school should introduce a course of sex education in the curriculum. Science teachers could be responsible for teaching these courses. However, medical doctors specialized in sexually transmitted diseases (gynecologists, urologists, dermatologists), having both a theoretical knowledge and a practical experience on these topics, would be the most appropriate figures to instruct teenagers (and also teachers) about the risks related to unprotected sexual practice, smoking, use of alcohol and drugs of abuse. An exam at the end of the course would be useful to check if and how students have acquired the knowledges, as well as they do for the other teachings. Moreover, it would be helpful to establish in each school a medical service dedicated to sex education and informations on sexually transmitted diseases, as it exists, in some schools, the psychological support service run by a psychologist.

Finally, we believe that only increasing the awareness on these topics starting from the school age, young people may understand the right behaviors to adopt for living a healthier life in the interests of themselves and of the entire society.

## Figures and Tables

**Table 1:** Results of the first section of the questionnaire.

Question	Answers (%)
What is the role of school and parents in sex education and prevention of STDs?	School should play the primary role (95%); parents should play the primary role (5%)
Sexual education received at school has been considered:	Good (9%); sufficient (32%); insufficient (36%); non-existent (23%)
Sources of information about sex:	Parents and teachers (37%); friends (25%); internet (15%); books (16%); 7% of the students did not specify sources of information
Sexual education determines:	Better awareness and reassurance (90%); anxiety and discomfort (7%); both (3%)
Do you feel sufficiently informed to avoid risk of STDs transmission?	Yes (53%); no (13%); I don‘t know (34%)
Communication with parents is:	Excellent (27%); good (43%); moderate (24%); non-existent (5%)

**Table 2:** Results of the second section of the questionnaire.

Question	Correct Answers (%)	Wrong Answers (%)	
Which of the following diseases are sexually transmitted? HIV, syphilis, hepatitis A, hepatitis B, hepatitis C, herpes simplex infection, candida and genital warts	All these infections, except for hepatitis A (0.5%)	95% of the students did not recognize these diseases as sexually transmitted	
How can you acquire a STI?	“Having sexual intercourse with any partner “or“ even through a single sexual intercourse” (15%)	“Only with prostitutes“, “only with homosexual relatons“, “only with repeated reports“, “only with occasional reports“(85%)	
Can you acquire an STI through orale sex?	Yes (42%)	No (23%)	I don‘t know (35%)
Which means of contraception (intrauterine device, contraceptive pill, trans-dermal patch, vaginal ring, condoms, spermicidal gel, *coitus interruptus*, natural methods, abstinence) protect against STDs?	Condoms and/or abstinence (22%)	One or more of the other methods (78%)	
Which are the body fluids at high risk for HIV transmission?	Blood, spermatic fluid and vaginal secretions (26%)	Urine, saliva, sweat (74%)	
Would you eat in a restaurant where an HIV positive man works?	Yes (46%)	No (54%)	
Do you know what a pap test is?	Yes (46%)	No (54%)	
Does a negative pap test assure a girl that she has not contracted any sexually transmitted disease?	No (23%)	Yes (29%)	I don‘t know (48%)
Which of the following infections between syphilis, candidiasis, HPV are potentially carcinogenic?	HPV (32%)	Syphilis or candida or none of these (68%)	
In case of HPV infection, the risk of developing cancer is higher in males, females or equal?	Females(43%)	Males (57%)	
Is there a vaccine that can protect against some types of STDs?	Yes (28%)	No (9%)	I don‘t know (63%)
If yes, what kinf of vaccine exists?	HPV vaccine (17%); HBV vaccine (0.1%)	Syphilis (0.3%), tetanus (0.1%), HCV vaccine (0.1%); no vaccines available (82.4%)	
What kind of tests are useful to diagnose a STD?	Blood tests (63%)	STDs are not diagnostiable (7%)	I don‘t know (24%)
Which doctor would you consult in the suspicion of an STD?	General Practitioner (26%), counseling physician (24%), gynaecologist (49%), dermatologist/first Aid physician/pharmacist (1%)		
Can you always heal from STDs?	No (46%)	Yes (4%)	I don‘t know (50%)

**Table 3:** Results of the third section of the questionnaire.

Question	Answers (%)
Influence of friends on the first sexual intercourse is:	great 16%, moderate 46%, little 26%, none 12%
Have you ever talked with their friends about HIV infection?	yes 47%, no 53%
Have you ever talked with you parents about HIV infection?	yes 45%, no 55%
If your partner asked you, would you do a HIV test?	yes 68%, no 6%, I don‘t know 25%
Have you ever had full sexual intercourses?	yes 61%, no 49%
If yes, at what age did you have your first full sexual intercourse?	mean age 15.5 ± 1.5 years
Did you use a contraceptive method during the first intercourse?	yes 77%, no 23%
If yes, which method did you use?	condom 93%, contraceptive pill 7%, both 3%
Are you currently using any method of prevention?	condom 37%, contraceptive pill 6%, condom + contraceptive pill 2%, other methods 13%, none 42%
If you use a condom, what is the reason?	as a method of contraception and to prevent STDs (100%)
If you do not use a condom what is the reason?	use of other contraceptive methods as pill (92%); concern about losing spontaneity during the intercourse (8%)
With how many partners did you have full sexual intercourses in the last 2 years?	one (37%), two (11%), more than two (15%), nobody (34%)
What kind of partners have you had?	steady partner (47%), steady partner+occasional partner (6%), only occasional partners (10%), no answer (37%)
Have you ever used alcoholic drinks ?	yes 79%, no 21%
On what occasions do you use alcoholic drinks?	parties 59%, weekend 24%, during the week 2%, never 15%
Have you ever use drugs of abuse?	yes 30% (especially cannabis but also LSD, ecstasy, cocaine), no 70%
Do you believe that sex education and STD prevention could improve the quality of life?	very much 68%, fairly enough 25%, little 4%, not at all 2%

## Supplementary Materials

The following are available online at www.mdpi.com/1660-4601/13/4/422/s1, Figure S1: Details about sources of sexual information.
