# Supplementary material for: A Survey of Current Knowledge on Sexually Transmitted Diseases and Sexual Behaviour in Italian Adolescents
Source: Int J Environ Res Public Health. 2016 Apr 13;13(4):422. doi: 10.3390/ijerph13040422 (PMC4847084; doi:10.3390/ijerph13040422)
Supplement: Supplementary file 1 [file ijerph-13-00422-s001.pdf]

Supplementary Materials: A Survey of Current Knowledge on Sexually Transmitted Diseases and Sexual Behaviour in Italian Adolescents

Francesco Drago, Giulia Ciccarese, Francesca Zangrillo, Giulia Gasparini, Ludovica Cogorno, Silvia Riva, Sanja Javor, Emanuele Cozzani, Francesco Broccolo, Susanna Esposito and Aurora Parodi

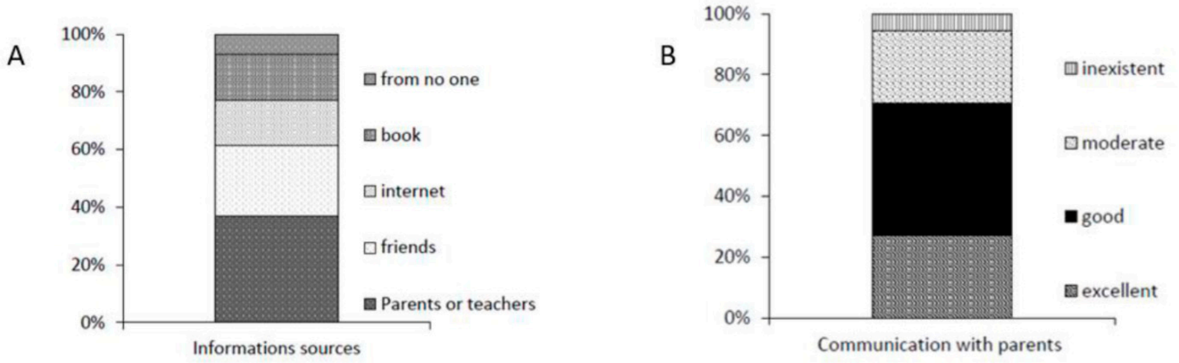

Figure S1. Details about sources of sexual information. (**A**) In 37% of cases information about sex came from parents and teachers, in 25% from friends, in 15% from the internet, in 16% from books and 7% of the students did not want to specify the source of their information; (**B**) Communication with parents was considered excellent by 27% of the students, good by 43%, moderate by 24%, and non-existent by 5%.

© 2016 by the authors; licensee MDPI, Basel, Switzerland. This article is an open access article distributed under the terms and conditions of the Creative Commons by Attribution (CC-BY) license (http://creativecommons.org/licenses/by/4.0/).
